# The Potential Role of Sanguinarine as an Inhibitor of *Leishmania* PP2C in the Induction of Apoptosis

**DOI:** 10.1007/s11686-024-00977-x

**Published:** 2025-01-24

**Authors:** M. Ornelas-Cruces, A. R. Escalona-Montaño, N. Salaiza-Suazo, S. Sifontes-Rodríguez, M. M. Aguirre-García

**Affiliations:** 1 Laboratorio de Estudios Sociales de la Ciencia y la Tecnología, Departamento de Biología Evolutiva, Facultad de Ciencias, Universidad Nacional Autónoma de México, Ciudad de México, México; 2 División de Investigación, Facultad de Medicina, Unidad de Investigación UNAM-INC, Universidad Nacional Autónoma de México, Instituto Nacional de Cardiología Ignacio Chávez, Juan Badiano No. 1, Col. Sección XVI, Ciudad de México, C.P. 14080 México; 3 Facultad de Medicina, Unidad de Investigación en Medicina Experimental, Universidad Nacional Autónoma de México, Avenida Universidad 3000, Ciudad de México, C.P. 04510 México

**Keywords:** Apoptosis, *Leishmania mexicana*, *Leishmania major*, PP2C, Sanguinarine, Protozoan

## Abstract

*Leishmania* spp. cause a wide range of human diseases, localized skin lesions, mucocutaneous and visceral infections. In the present study, the aim was to investigate the potential role of sanguinarine as a specific inhibitor of *Leishmania* PP2C that can induce apoptosis in the parasite. The results demonstrated that sanguinarine inhibits, in a dose-dependent mode at 72 h, the growth and phosphatase activity of both *Leishmania major* and *Leishmania mexicana* promastigotes. Therefore, all assays were performed from this time period onwards. TUNEL assay was used to identify apoptosis and indicated apoptosis in *L. major* and *L. mexicana* promastigotes. Similarly, Western blot assay showed that PARP, a DNA damage indicator molecule, was present in *L. major* and *L. mexicana* promastigotes incubated with the inhibitor. In addition, differential expression of the proapoptotic protein Bax and the antiapoptotic protein Bcl-2 was observed in both *Leishmania* species. Finally, the protein phosphatase PP2C expression was not affected, whereas p38 MAPK phosphorylation was increased in *L. major* promastigotes than in *L. mexicana* promastigotes. Therefore, sanguinarine proved to be an inhibitor of the growth and PP2C enzymatic activity of *L. major* and *L. mexicana* promastigotes, and with it, this inhibition induced apoptosis.

## Introduction

Apoptosis is a type of programmed cell death that actively participates in the development of multicellular organisms. In general, the cell that enters the programmed death process performs all its functions normally and when apoptosis is triggered, it dies without releasing its contents to the surrounding environment. Apoptosis can be initiated by two pathways: the first is initiated by the cell itself when it enters into stress conditions and detects damage through a signalling cascade; this mechanism is known as the mitochondrial intrinsic apoptosis pathway. In contrast, the second pathway can result from the interaction between death receptors and death ligands, and it is known as the death receptor or extrinsic apoptosis pathway [1].

The intrinsic apoptosis pathway is regulated by the balance between pro- and anti-apoptotic members of the B-cell lymphoma-2 (Bcl-2) family of proteins, and typically involves DNA fragmentation, activation of endonucleases, destruction of the nuclear proteins and the cytoskeleton, formation of apoptotic bodies, disruption of mitochondrial outer membrane permeability, release of cytochrome c from mitochondria into the cytosol, and activation of the caspase cascade via caspase-9. One of the domains of Bcl-2, particularly BH4, is able to bind to other proteins that do not belong to the Bcl-2 protein family, allowing them to perform other functions -in addition to the classical one of inhibiting apoptosis- such as proliferation, autophagy, differentiation, DNA repair, tumour progression and angiogenesis [2].

Other protein members of the Bcl-2 family are important regulators of the mitochondrial apoptosis pathway. These members are inducers of the cell death process and one of the classical proapoptotic members is Bax (Bcl-2-associated protein X), which induces cell death by homodimerisation and heterodimerisation with Bcl-2 [3]. In addition, mitogen-activated protein kinases (MAPK) enzymes are serine-threonine kinases that have been classified into three main subfamilies of which include p38 (p38 MAPK) and it responds to both stress and intracellular signals. For these reasons, it has been studied and shown that the p38 MAPK signalling cascade regulates apoptosis [4].

The aforementioned is classic for multicellular organisms, especially mammals, where apopotosis has been extensively studied, with emphasis on humans and for disease research purposes. There is now increasing experimental evidence that suggests that a similar form of cell death is operative in unicellular eukaryotes, including trypanosomatids of the genus *Leishmania* [5]. *Leishmania* spp. are intracellular parasites that infect macrophages and other cells of the reticuloendothelial system, and they can modulate signaling pathways of host cells [6, 7].

Apoptosis in these trypanosomatids is induced by various stimuli such as heat shock, reactive oxygen species, starvation, antiparasitic drugs such as miltefosine, and exhibits typical features of programmed cell death, including cell shrinkage, nuclear condensation, and DNA fragmentation [8–11]. *Leishmania* spp. modify host cell signaling by regulating intracellular MAPKs and the janus kinase (JAK)-signal transducer/activator of transcription (JAK/STAT)-mediated signaling [12]. The mechanisms by which this modification of the signaling takes place is through phosphorylation and dephosphorylation, which generally occurs on serine/threonine residues [13, 14].

The enzymes that perform this function of dephosphorylation are called phosphatases, and these are classified into the serine/threonine phosphatases (STPs) which have three families of molecules, one of which is the phosphoprotein phosphatases (PPP). The PPP family consists of the PP1, PP2A and PP2B phosphatases, as well as the metal-dependent protein phosphatases (PPM) either Mg^2+^ or Mn^2+^. Among the PPMs is protein phosphatase type 2 C (PP2C) [12, 15–17].

Some studies suggest that PP2C of different pathogens such as *Cryptosporidium parvum*, *Plasmodium falciparum* and *Toxoplasma gondii* play a critical role in the pathogenesis of these parasites [15, 17–19]. There are many examples of transcriptional control of PP2C expression in plants, which include stress response, abscisic acid signaling, and development [20, 21]. Also, this protein has been related to cell death because PP2C dephosphorylates several intracellular substrates like cyclin-dependent kinase, MAPK and signaling-related proteins in apoptosis such as the Bcl2-associated agonist of cell death (BAD, a pro-apoptotic protein) [22].

Other studies have reported the existence of a PP2C inhibitor known as sanguinarine, which is an alkaloid obtained from the bloodroot plant *Sanguinaria canadensis*. This inhibitor induces apoptosis or inhibits formation and growth of human promyelocytic leukemia cell line HL-60. Nevertheless, the mechanism of action of sanguinarine is still unknown even in trypanosomatids [22–24]. Escalona Montaño et al., has cloned and characterized the PP2C of *Leishmania major* and *Leishmania mexicana* and these proteins have biochemical properties similar to those of PP2C of other eukaryotes [16, 25]. The aim of this study was to determine whether sanguinarine, by inhibiting PP2C, induces apoptosis in *L. major* and *L. mexicana* promastigotes.

## Materials and methods

### Preparation of Sanguinarine

Sanguinarine (Sigma-Aldrich, ST Louis, MO, USA) was dissolved in methanol to a final concentration of 13.5 mM.

### Parasite Culture

The Friendlin strain of *Leishmania major* was donated by Dr. Santiago Martinez Calvillo (Unidad de Investigación en Biomedicina, Facultad de Estudios Superiores Iztacala, UNAM, Estado de México) and the MNYZ/BZ/62/M379 strain of *Leishmania mexicana* was donated by Dr. Paul Bates (Lancaster University, Lancaster, UK). Both strains were cultured at 26 °C in medium 199, pH7.2 supplemented with 10% fetal bovine serum (FBS), 1% antibiotic Penicillin-Streptomycin (penicillin 10, 000 Units/mL and streptomycin 10, 000 ug/ mL) and 1% basal medium Eagle (BME) vitamins (all from Gibco of Thermo Fisher Scientific, Waltham, MA, USA). The parasites were passaged in log phase.

To preserve the virulence of the strains, 10^7^ stationary-phase promastigotes were inoculated into the footpad of BALB/c mice. Once lesions developed, parasites were isolated according to a methodology described elsewhere [26] with some modifications. Mice were sacrificed by cervical dislocation and the infected foot was excised, which was macerated with phosphate buffer solution (PBS) in a 10 mL syringe plunger on a nylon mesh of 100 μm pores (BD Falcon, Bedford, MA, USA). The obtained suspension was centrifuged at 100 g for 5 min at room temperature. The supernatant was recovered and centrifuged at 2000 g for 10 min to obtain the pellet corresponding to the amastigotes. They were washed three times with PBS and cultured in the medium described above at 26 °C to achieve differentiation into promastigotes. Every day, at 72 h, a sample of parasite culture was taken; parasite promastigotes were immobilized by dilution in paraformaldehyde (Sigma-Aldrich, ST Louis, MO, USA) and counted using a Neubauer chamber. Morphology was analyzed by phase-contrast microscopy of the same samples.

### Exposure of Promastigotes to Sanguinarine

Exponentially growing cultures of *L. major and L. mexicana* were adjusted in fresh culture medium to 5 × 10^5^ parasites/mL in a final volume of 10 mL and incubated for 72 h at 26 °C in the presence of 50, 100 and 250 nM of sanguinarine.

### Total Extracts of Promastigotes

After 72 h of incubation of the promastigotes in media containing 50, 100 and 250 nM concentrations of sanguinarine, the parasites were washed three times with PBS by centrifugation at 2000 g for 10 min at room temperature. The pellet obtained after the final wash was stored at -70 °C until use. Then, the pellet was resuspended in a lysis buffer made up of 50 mM sterile buffer Tris-HCl, pH 7.4 and a cocktail of protease inhibitors (leupeptin 0.002 µg/mL, aprotinin 0.01 µg/mL and 1mM benzamidine). Buffer and protease inhibitors were from Sigma-Aldrich, ST Louis, MO, USA The resuspended pellet was lysed using a sonicator (Sonics, Vibracell™ Newtown, CT, USA) by a cycle of 1 min at 4 °C and an amplitude of 30%. Parasite extracts obtained this way were stored at -70 °C until use.

### Phosphatase Activity Assays

In a 96-well plate, a reaction containing 0.2 M sodium acetate was obtained from J.T. Baker (Center Valley, PA, USA), parasite extract (10 µg of protein from the total extract) and 10 µL of 0.1 M phosphatase substrate *p*-nitrophenylphosphate (*p*-NPP) (Sigma-Aldrich, ST Louis, MO, USA) was added in a final volume of 100 µL/well. The reaction was incubated for 1 h at 37 °C and stopped with 20 µL of 2 N sodium hydroxide from J.T. Baker (Center Valley, PA, USA) before reading in a spectrophotometer (BiotekR, µQuant) at a wavelength of 405 nm.

### Apoptosis in Promastigotes by TdT-mediated dUTP-biotin nick end-labeling (TUNEL)

Promastigotes washed by the method described in 2.4 were adhered on a slide and fixed with 4% paraformaldehyde (Sigma-Aldrich, ST Louis, MO, USA) for 1 h at room temperature. Then, they were incubated for 10 min with 3% methanol from J.T. Baker (Center Valley, PA, USA) in order to block the activity of endogenous peroxidases. After washing the samples three times with PBS, they were treated with a permeabilizer solution (0.1% Triton X-100 in 0.1% sodium citrate, Sigma-Aldrich, ST Louis, MO, USA and J.T. Baker (Center Valley, PA, USA) respectively) on ice for 2 min. Again, the samples were washed three times with PBS and incubated with the enzyme solution of the TUNEL kit (In situ Cell Death Detection Kit; Roche Diagnostic GmbH, Mannheim, Germany) for 1 h in a humid chamber at room temperature. The slides were then, washed three times with PBS, and the peroxidase activity was revealed with aminoethylcarbazole and counterstained with hematoxylin. The samples were analyzed under the microscope AxioPlan II Zeiss (Zeiss, Thornwood, NY, USA) and the number of parasites with stained nucleous were determined on the basis of 100 parasites observed.

### Identification of poly-ADP-ribose Polymerase (PARP), Bcl-2 and Bax Proteins by Western blot

For the analysis of PARP and Bcl-2 protein an electrophoresis was performed using a 15% gel SDS-PAGE. 40 µg of the total promastigote extract described above were used. For the electrophoresis, a voltage of 70 V was applied for 30 min and then at 120 V until completion. After that, the gel was equilibrated in transfer buffer for 10 min and the transfer of proteins to a previously activated PVDF membrane (Millipore-Merck KGaA, Darmstadt, Germany) was performed in methanol for 3 min and washed three times with deionized water for 5 min each and balanced in transfer buffer. The PVDF membrane was placed in a semi-dry electroblotting chamber (Bio-Rad Laboratories, Hercules, CA, USA) at 20 V for 60 min. Membranes were blocked with 5% milk powder (Bio-Rad Laboratories) in TBST and washed with buffer Tris-Tween (TBST) six times every 10 min and incubated with the corresponding antibodies: for α−poly-ADP-ribose polymerase was used at a volume of 0.5 µL/mL (Roche Diagnostics GmbH, Mannheim, Germany) and both α−Bcl2 and α−Bax with a 1:1000 dilution (Santa Cruz Biotechnology, Inc., Dallas, TX, USA), α−PP2C at 1:1000 dilution, α−p38 MAPK at 1:500 (Cell Signaling Technology, Inc., Danvers, MA, USA) overnight in constant agitation at 4 °C. The membranes were washed six times with TBST every 10 min. The membranes were incubated for 1 h at room temperature with secondary antibody anti-mouse IgG-HRP and anti-rabbit IgG-HRP (Cell Signaling Technology Inc, Danvers, MA, USA) diluted in 5% milk powder in TBST with a 1:5000 dilution. After washing six times with TBST every 10 min, the membranes were developed with a chemiluminescent substrate for HRP (Millipore-Merck KGaA), and exposed to X-ray films (Santa Cruz Biotechnology, Inc. α−tubulin was used as loading control.

### Statistical Analyses

All the data were analyzed using GraphPad Prism version 9.0 (GraphPad Software, La Jolla California USA). Data are presented as mean ± S.D. The groups were compared using one-way analysis of variance (ANOVA) with Tukey’s post hoc test for multiple comparisons. Values of *p* < 0.05 were considered significant.

## Results

### Effect of Sanguinarine upon the Growth and Viability of Promastigotes from *L. Major* and *L. Mexicana*

**Fig. 1:**
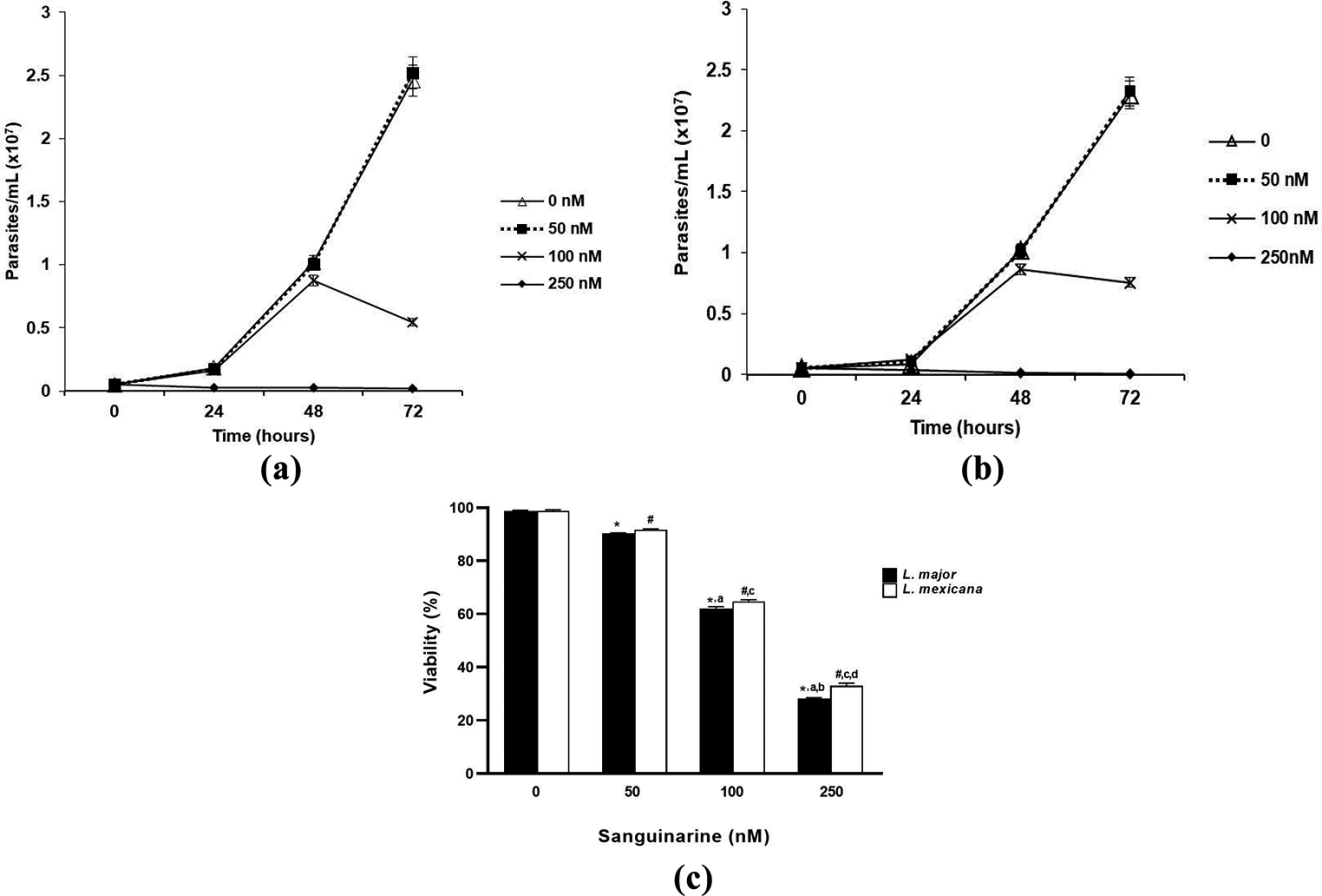
**A** Growth of *L*. *major* promastigotes incubated in media with sanguinarine at concentrations of 50, 100 and 250 nM for 72 h of culture and counted using Neubauer’s chamber. Graphs are representative of three independent experiments. **B** Growth of *L*. *mexicana* promastigotes incubated in media with sanguinarine at concentrations of 50, 100 and 250 nM for 72 h of culture and counted using Neubauer’s chamber. Graphs are representative of three independent experiments. **C** Viability of *L. major* and *L. mexicana* promastigotes stained with erythrosin B incubated in media with sanguinarine at concentrations of 50, 100 and 250 nM.at 72 h of culture The data are representative of three independent experiments. Values represented as mean ± S.D. ANOVA one way. *Post hoc* Tukey. *p < 0.0001 vs. 0. ^a^*p* < 0.0001 vs. 50. ^b^*p* < 0.0001 vs. 100 in *L. major.*^*#*^*p* < 0.0001 vs. 0. ^c^*p* < 0.0001 vs. 50. ^d^*p* < 0.0001 vs. 100 in *L. mexicana.n* = 3

We analyzed the growth of *L. major* and *L. mexicana* promastigotes in the absence and presence of 50, 100 and 250 nM of sanguinarine because, as previously reported, sanguinarine inhibits the activity of recombinant LmPP2C at a concentration of 20 µM [25]. After 72 h of culture, we found optimal growth of *L. major* promastigotes incubated without sanguinarine. Their cell density was 2.5 × 10^7^ cells/mL approximately. Similarly, promastigotes incubated in medium with sanguinarine at a concentration of 50 nM had almost the same cell density. In contrast, from 48 h of culture, promastigotes incubated in medium with sanguinarine at a concentration of 100 nM had a cell density of approximately 8 × 10^5^ cells/mL; and at 72 h of culture, the cell density was 5 × 10^5^ cells/mL. In addition, promastigotes incubated in medium with sanguinarine at a concentration of 250 nM did not exhibit growth from the first 24 h of incubation (Fig. 1A).

When we analysed *L. mexicana* promastigotes, we found that growth was remarkably similar among cultures without sanguinarine and those with a concentration of 50 nM, as the cell density for both was approximately 2.3 × 10^7^ cells/mL. However, after 48 h of culture, the promastigotes incubated in medium with sanguinarine at a concentration of 100 nM had a cell density close to 1 × 10^7^ cells/mL, and at 72 h of culture the cell density was approximately 9 × 10^5^ cells/mL. Additionally, promastigotes incubated in medium with sanguinarine at a concentration of 250 nM showed no growth from the first 24 h of incubation (Fig. 1B).

Vital dye staining of promastigotes with erythrosin B [27] showed that parasites incubated in medium with sanguinarine at a concentration of 50 nM at 72 h had a viability of approximately 90% for both *L. major* and *L. mexicana*. Parasites incubated in medium with sanguinarine at a concentration of 100 nM at 72 h had a viability of around 60% for both species, and those incubated in medium with sanguinarine at a concentration of 250 nM, had a viability of roughly 30% (Fig. 1C).

### Inhibition of Phosphatase Enzyme Activity upon the Presence of the Inhibitor Sanguinarine in Total Extracts of Promastigotes from *L. Major* and *L. Mexicana*

**Fig. 2:**
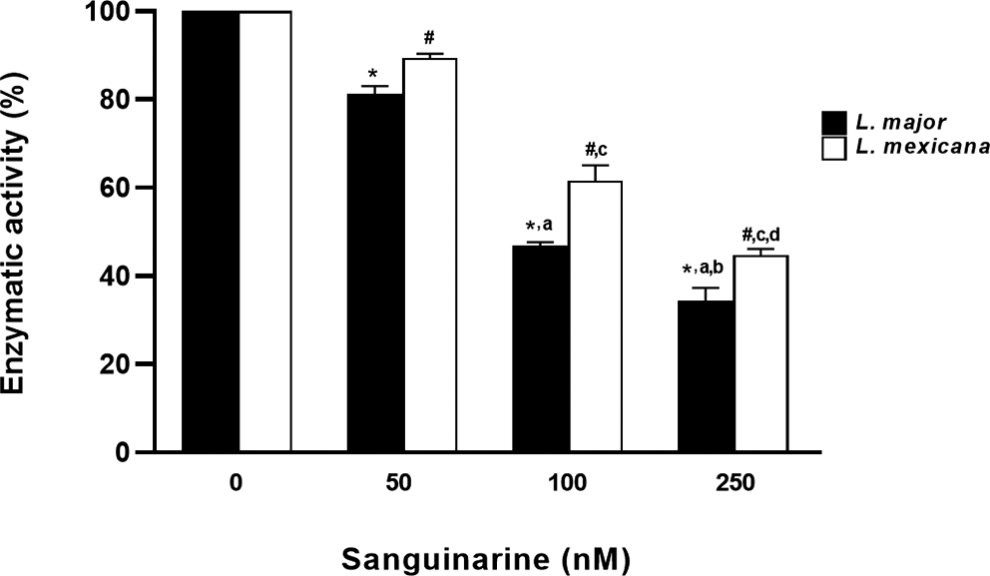
**A** Phosphatase enzyme activity graph in total extracts of *L. major* and *L. mexicana* promastigotes obtained by sonication. The data are representative of three independent experiments. Values represented as mean ± S.D. ANOVA one way. *Post hoc* Tukey. *p < 0.0001 vs. 0. ^a^*p* < 0.0001 vs. 50. ^b^*p* < 0.0001 vs. 100 in *L. major.*^*#*^*p* < 0.0001 vs. 0. ^c^*p* < 0.0001 vs. 50. ^d^*p* < 0.0001 vs. 100 in *L. mexicana*. *n* = 3

As for the enzyme activity of phosphatase, we observed at 72 h in all assays that it was approximately 90% for *L. major*, while for *L. mexicana* it was 80%, in both cases of parasites incubated in the media with sanguinarine at a concentration of 50 nM. Also, those incubated in media with sanguinarine at a concentration of 100 nM, the percentage of enzyme activity was 60% for *L. major*, while for *L. mexicana* it was 50%. Finally, parasites incubated in media with sanguinarine at a concentration of 250 nM, the enzyme activity was slightly higher than 40% for *L. major*, while for *L. mexicana* it it was slightly higher than 30%. (Fig. 2A).

### Effect of Sanguinarine on the Morphology of Promastigotes from *L. Major* and *L. Mexicana*

#### Morphology of Promastigotes

**Fig. 3:**
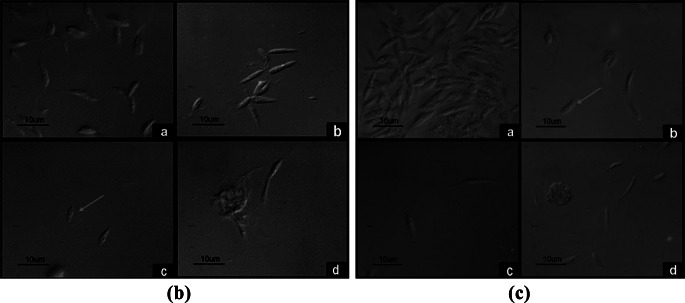
**B** Morphology of *L. major* promastigotes incubated in media with sanguinarine at 72 h of culture and analyzed by phase-contrast microscopy. The red arrow indicates a parasite without a flagellum. Concentrations were **a**: no inhibitor, **b**: 50 nM, **c**: 100 nM, **d**: 250 nM. Micrographs are representative of three independent experiments. **C** Morphology of *L. mexicana* promastigotes incubated in media with sanguinarine at 72 h of culture and analyzed by phase-contrast microscopy. The red arrow indicates a parasite without a flagellum. Concentrations were **a**: no inhibitor, **b**: 50 nM, **c**: 100 nM, **d**: 250 nM. Micrographs are representative of three independent experiments

Regarding the effect of sanguinarine on the morphology of *L. major* parasite promastigotes, at 72 h of the experiment, we observed that when incubated without sanguinarine, the parasites did not show morphological **(**Fig. 3Ba). Similarly, for promastigotes incubated in medium with sanguinarine at a concentration of 50 nM, we found that although the morphology was similar to parasites incubated without sanguinarine (Fig. 3Bb). In the case of promastigotes incubated in medium with sanguinarine at a concentration of 100 nM, we observed that some parasites showed an absence of the flagellum and a reduced size compared to parasites incubated without sanguinarine (Fig. 3Bc). Finally, promastigotes incubated in medium with sanguinarine at a concentration of 250 nM showed a great deterioration in morphology to the extent that most of them were in the process of cell lysis (Fig. 3Bd).

In the case of *L. mexicana* promastigotes incubated without sanguinarine, at 72 h of the experiment, they did not showed changes in morphology (Fig. 3Ca). Meanwhile, promastigotes incubated in medium with sanguinarine at a concentration of 50 nM showed variations in size (Fig. 3Cb). Promastigotes incubated in medium with sanguinarine at a concentration of 100 nM showed no differences in morphology. (Fig. 3Cc). However, parasites incubated in medium with sanguinarine at a concentration of 250 nM were deformed and almost all promastigotes lacked a flagellum (Fig. 3Cd).

### Analysis of Apoptosis in Promastigotes of *L. Major* and *L. Mexicana*

#### Analysis of DNA Fragmentation by TUNEL

**Fig. 4:**
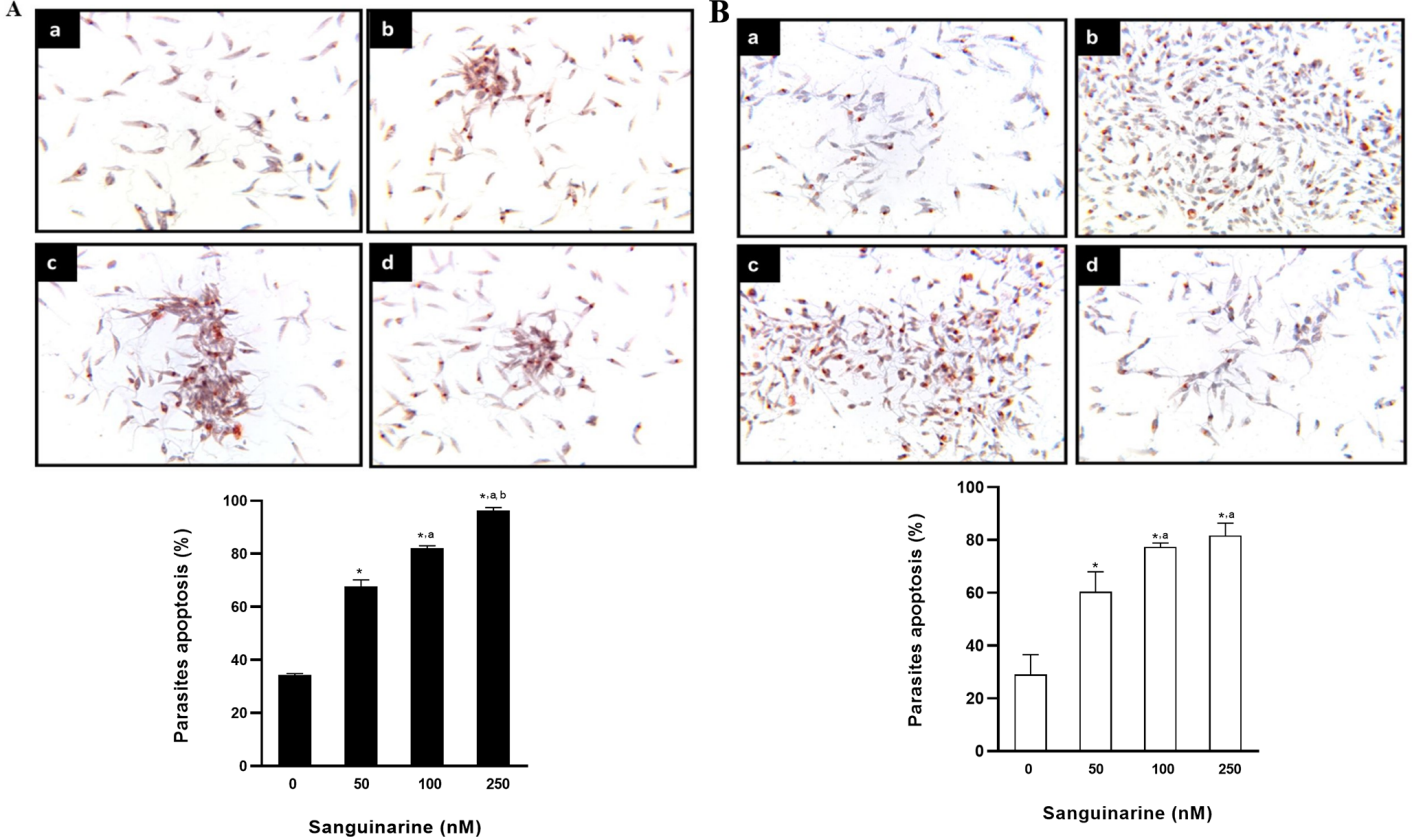
**A** DNA fragmentation in promastigotes of *L. major* incubated in media with sanguinarine at 72 h. Micrographs of *L. major* promastigotes showing DNA fragmentation by TUNEL technique. The images show the stained nuclei under the different experimental conditions. Concentrations were a: no inhibitor, b: 50 nM, c: 100 nM, d: 250 nM. All data are representative of three independent experiments. Values represented as mean ± S.D. ANOVA one way. *Post hoc* Tukey. *p < 0.0001 vs. 0. ^a^*p* < 0.0001 vs. 50. ^b^*p* < 0.0001 vs. 100. *n* = 3. **B** DNA fragmentation in promastigotes of *L. mexicana* incibated in media with sanguinarine at 72 h. Micrographs of *L. mexicana* promastigotes showing DNA fragmentation by TUNEL technique. The images show the stained nuclei under the different experimental conditions. Concentrations were a: no inhibitor, b: 50 nM, c: 100 nM, d: 250 nM. All data are representative of three independent experiments. Values represented as mean ± S.D. ANOVA one way. *Post hoc* Tukey. *p < 0.0001 vs. 0. ^a^*p* < 0.0001 vs. 50. ^b^*p* < 0.0001 vs. 100. *n* = 3

DNA fragmentation in promastigotes of both *L. major* and *L. mexicana* was analysed by TUNEL at 72 h of culture in the absence and presence of sanguinarine. The former, incubated in medium without sanguinarine, showed approximately 30% basal apoptosis. Then, we observed that promastigotes incubated in medium with sanguinarine at a concentration of 50 nM showed about 65% apoptosis. Subsequently, we observed that promastigotes incubated in medium with sanguinarine at a concentration of 100 nM showed about 80% apoptosis. Finally, we observed that promastigotes incubated in medium with sanguinarine at a concentration of 250 nM showed about 90% apoptosis (Fig. 4A). The promastigotes of *L. mexicana* incubated in medium without sanguinarine, showed approximately 35% basal apoptosis. Then, we observed that promastigotes incubated in medium with sanguinarine at a concentration of 50 nM showed about 70 to 75%. Later, we observed that promastigotes incubated in medium with sanguinarine at a concentration of 100 nM showed about 80% apoptosis. Finally, we observed that promastigotes incubated in medium with sanguinarine at a concentration of 250 nM showed 90 to 100% apoptosis (Fig. 4B).

#### Identification of PARP in Total Extracts

**Fig. 5:**
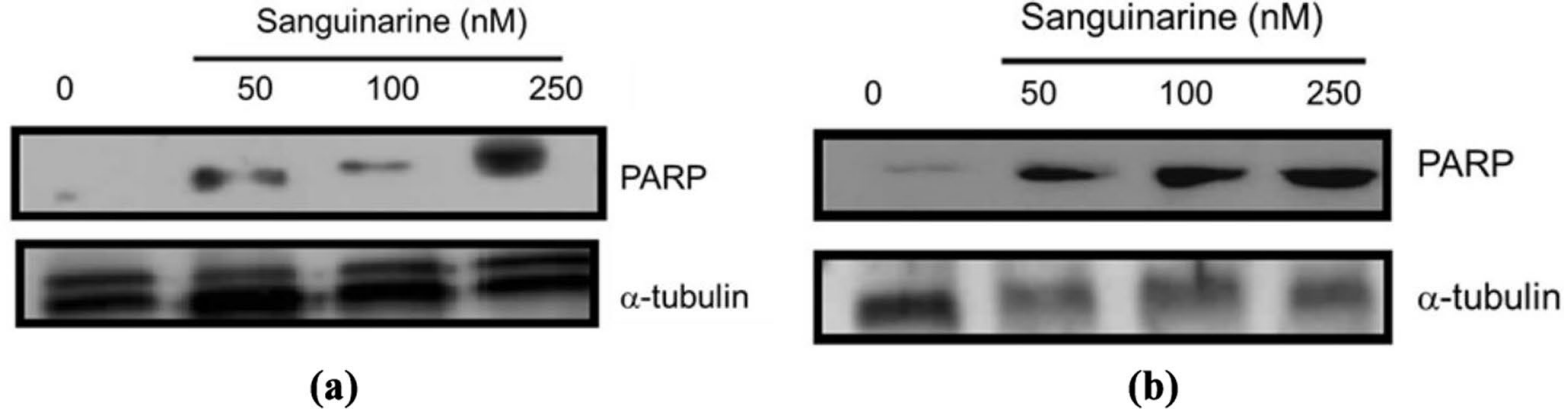
**A** Total extracts of *L. major* promastigotes incubated in media with sanguinarine at 50, 100 and 250 nM were used to identify DNA damage-related proteins. **B** Total extracts of *L. mexicana* promastigotes incubated in media with sanguinarine at 50, 100 and 250 nM were used to identify DNA damage-related proteins

Western blot analyses showed an increased in PARP protein identification on total promastigote extracts at 72 h of culture of both *L. major* and *L. mexicana* in the absence and presence of sanguinarine. In the former we observed that promastigotes incubated in medium with sanguinarine at concentrations of 50 and 100 nM, PARP protein was identified. However, the identification of the PARP protein in total extracts of promastigotes incubated in medium with sanguinarine at a concentration of 250 nM is more discernible (Fig. 5A). On the contrary, in total extracts of *L. mexicana* promastigotes we observed that PARP protein were identified in all the experimental conditions (Fig. 5B).

#### Identification of pro-apoptotic Protein Bax, anti-apoptotic Protein Bcl-2, PP2C and p38 MAPK

**Fig. 6:**
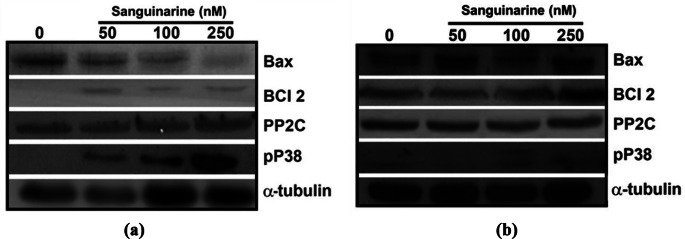
**A** Identification of pro-apoptotic protein Bax, anti-apoptotic protein Bcl-2, PP2C and p38 MAPK in total extracts of *L. major* promastigotes incubated in media with sanguinarine at 50, 100 and 250 nM. We used α-tubulin as a loading control in all experiments. **B** Identification of pro-apoptotic protein Bax, anti-apoptotic protein Bcl-2, PP2C and p38 MAPK in total extracts of *L. mexicana* promastigotes incubated in media with sanguinarine at 50, 100 and 250 nM. We used α-tubulin as a loading control in all experiments

We identified the pro-apoptotic protein Bax in total promastigote extracts at 72 h of culture of both *L. major* and *L. mexicana* in the absence and presence of sanguinarine at 50, 100 and 250 nM. In the former, we observed that the identification of protein Bax decreased as the concentration of sanguinarine increased. Nevertheless, the anti-apoptotic protein Bcl-2 was identified only in the total extracts of *L. major* parasites incubated in media with sanguinarine at concentrations of 50, 100 and 250 nM. Also, the PP2C was identified with and without sanguinarine in total *L. major* promastigotes extracts. However, we observed the phosphorylation of p38 MAPK only in total extracts of *L. major* parasites incubated in media at the concentrations of 50, 100 and 250 nM (Fig. 6A). We also identified the pro-apoptotic protein Bax in total promastigote extracts at 72 h of culture of *L. mexicana* in the absence and presence of sanguinarine. We observed bands of protein Bax, of the anti-apoptotic protein Bcl-2, and PP2C in the phosphorylated state, in all experimental conditions. However, we observed that phosphorylation of p38 MAPK in total extracts of parasites incubated in media at concentrations of 100 and 250 nM (Fig. 6B).

## Discussion

*Toxoplasma gondii* and Trypanosomatids, in general, and *Leishmania* spp. in particular, regulate host cell apoptosis in their favor to invade it. However, the mechanism of apoptosis in protozoan parasites is not very clear and has even been referred to as apoptosis-like [5, 8, 9, 28, 29].

Although the mechanism of apoptosis in mammals is well known, key apoptotic proteins such as caspases, cell death receptors and anti-apoptotic molecules are not entirely present in *Leishmania* genus. Recent studies have contributed to develop a better understanding of parasite cell death, identifying new proteins, conserved motifs, and new apoptotic pathways [30].

Apoptosis-associated proteins, such as PP2C, have been extensively studied in plant models like *Arabidopsis* sp. and *Zea mays* (common maize). In these cases, PP2C is related to plant stress, hormone production (such as abscisic acid synthesized in leaves, stems, roots and green fruits) and to the dephosphorylation of the MAPK signaling cascade [20, 31, 32].

In addition, it has been reported that the inhibitor of PP2C known as sanguinarine, an extract from the plant *Sanguinaria canadensis*, has multiple properties such as antimicrobial, antifungal and antiprotozoal activity. It also blocks some cellular functions, including cell growth, and promotes the activation of p38 MAPK in HL-60 cells, through inhibition of PP2C, to induce apoptosis [22, 23, 33–35]. Sanguinarine also showed protective effect of against acetic acid-induced ulcerative colitis in mice [36] and its properties in primary efussion lymphoma cell [37].

In this context, our results showed, with respect to phosphatase activity, dose-dependent inhibitory curves in both *Leishmania* species incubated in media with sanguinarine under all experimental conditions. This was also analysed by Escalona-Montaño [16, 25] in recombinant *Lm*PP2C and is consistent with what is reported here. With regard to viability, we also observed dose-dependent curves in both *L. major* and *L. mexicana*, although in a differential way between the two species. Apparently, *L. mexicana* seems to be more resistant to sanguinarine than *L. major*. Also, the effect of sanguinarine on the morphology of the parasite allowed us to observe that it affects the shape of promastigotes of both *Leihsmania* species. With these results, we can suggest that PP2C is involved in the morphological alteration of the parasite, but it would be necessary to analyse the specific enzymatic activity of PP2C.

Regarding the growth of *L. major* and *L. mexicana* promastigotes incubated in media at a concentration of 50 nM was very similar to that in the absence of the inhibitor at 72 h of culture. However, the growth of those incubated in media at the concentration of 100 nM and at 48 h of culture was affected in a way that it decreased in comparison with the previous conditions. The most drastic effect was observed at 250 nM, since promastigotes growth was absent. These results confirmed what has been reported in other organisms, such as *Entamoeba* spp [38].

Using the TUNEL technique, it was observed that sanguinarine induced promastigotes to apoptosis from a concentration of 50 nM. We reached this claim by observing consistency in the canonical traits of apoptosis such as nuclear DNA fragmentation. Also, with the identification of PARP in both species of *Leishmania*, we observed that the higher the concentration of sanguinarine the higher the expression of the protein. It is important to note that PARP is activated in cells that are under stress conditions or with DNA damage and induces programmed cell death via a caspase-independent pathway, but the mechanism is not completely understood. These experiments support research on apoptosis in the genus *Leishmania* [30, 39].

Finally, we identified classical mammalian apoptosis proteins such as Bax and Bcl-2 in both *L. major* and *L. mexicana*. We observed in *L. major* under all experimental conditions, the pro-apoptotic protein Bax was present but its expression decreased with the concentration of sanguinarine. The anti-apoptotic protein Bcl-2 was only observed in the total extracts of parasites incubated in media with sanguinarine. We would like to highlight the presence of PP2C, whose relevance in this work could suggest a modulation in the apoptosis of promastigotes of both *Leismania* species. This could indicate that either PP2C is being activated by another unknown pathway, that sanguinarine is inhibiting PP2Cα and phosphorylating p38 MAPK [22], or that PP2Cβ is modulating apoptosis [24] of both *Leishmania* species. This could be associated with intracellular relationships with the host and even with the parasite strains themselves [40].

**Fig. 7:**
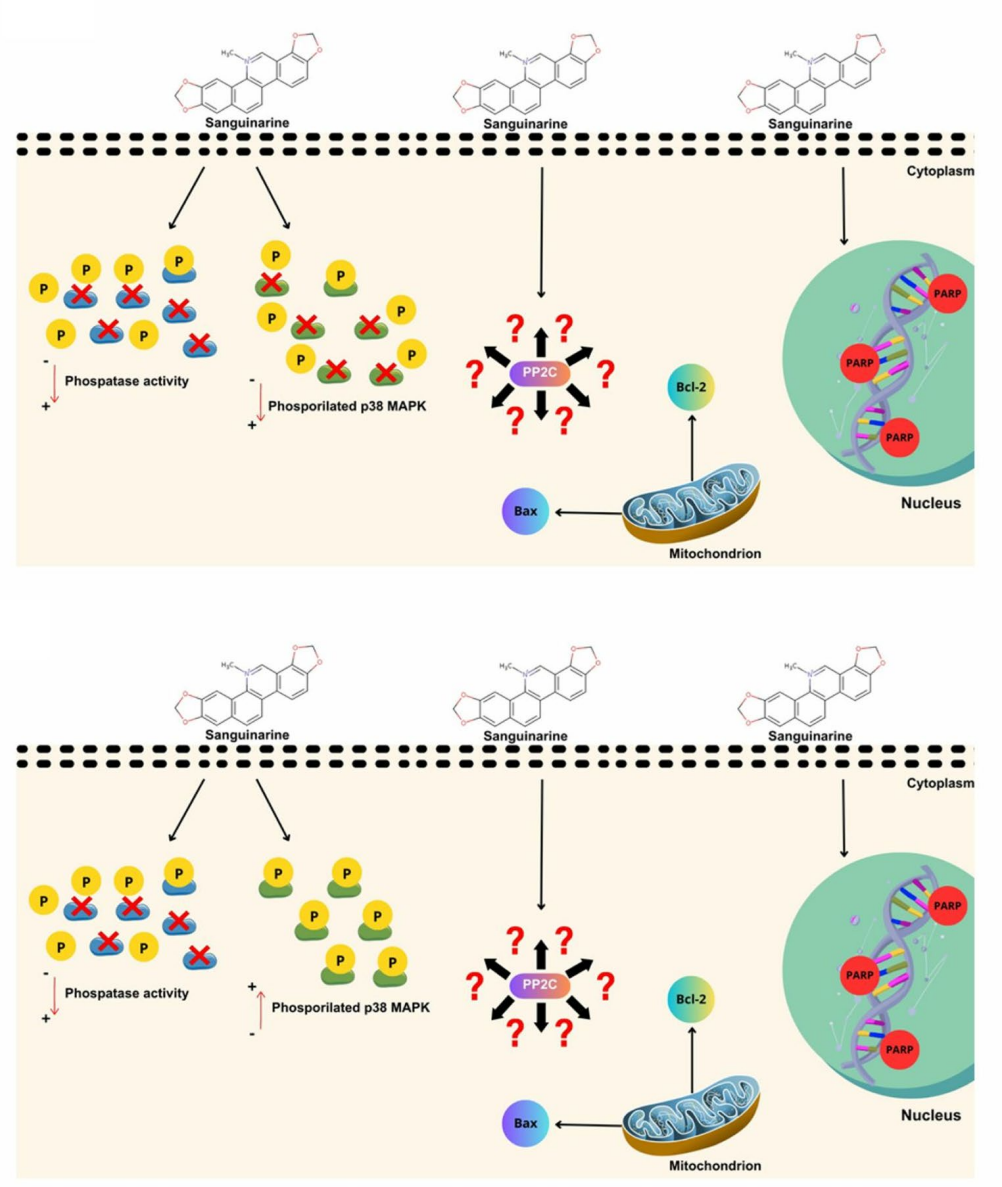
**A** Schematic illustration of the possible functions of PP2C-type phosphatase by interaction with the inhibitor sanguinarine in *L. major* and *L. mexicana*. The intrinsic pathway of apoptosis in *L. major* (upper) and *L. mexicana* (lower) is not yet fully understood. Evidence from our study shows that there may exist a correlation between the inhibitor sanguinarine and PP2C that induces apoptosis, despite differences in p38 MAPK phosphorylation in promastigotes of both *Leishmania* species

Therefore, due to the previous experimental evidence and what we are reporting in the present paper, we suggest that sanguinarine is possibly inhibiting PP2C of the parasite and, consequently, inducing apoptosis in *L. major* and *L. mexicana* promastigotes. Studies focused on exclusively inhibiting PP2C with sanguinarine could ensure that this phosphatase does modulate the intrinsic pathway of apopotosis in this parasite. In addition, we propose a scheme of the effects of sanguinarine and the way in which we assume that a PP2C-type phosphatase is inhibited by this compound. Even though we do not know the pathway completely, the results showed that there is apoptosis in *L. major* and *L. mexicana* promastigotes (Fig. 7A).

## Data Availability

No datasets were generated or analysed during the current study.
